# Nutrients and phytoplankton dynamics in the fishing grounds off Tiruchendur coastal waters, Gulf of Mannar, India

**DOI:** 10.1186/s40064-016-3058-8

**Published:** 2016-08-24

**Authors:** J. Selvin Pitchaikani, A. P. Lipton

**Affiliations:** 1 Centre for Marine Science and Technology, Manonmaniam Sundaranar University, Rajakkamangalam, Tamil Nadu India; 2 Vizhinjam Research Centre, Central Marine Fisheries Research Institute, Vizhinjam, Kerala 629 251 India; 3 Integrated Coastal Zone Management (ICZM) Project, Institute of Environmental Studies and Wetland Management (IESWM), DD-24, Sector-1, Salt Lake City, Kolkata, West Bengal 700 064 India

**Keywords:** Population abundance, Nutrients, Fishing grounds, Diatoms, Gulf of Mannar

## Abstract

Nutrients and phytoplankton dynamics in the traditional fishing grounds off Tiruchendur coast, Gulf of Mannar, India revealed a clear seasonal trend influenced by prevailing monsoon system in east coast of India. A total of 73 species of phytoplankton were identified from the fishing grounds, revealed higher abundance in summer months compared to other seasons. Among the three stations, maximum phytoplankton abundance was recorded in station 2 followed by stations 1 and 3. The phytoplankton abundance ranged from 2.85 × 10^4^ to 6.34 × 10^4^ cells/l, with higher and lower value observed during summer and post monsoon season respectively. Chl-a showed similar seasonal trend with phytoplankton abundance and fluctuated from 0.4 to 6.8 mg/m^3^ with high concentrates were recorded during summer. Primary productivity was ranged from 13.8 to 28.7 mg, C/m^2^/day with maximum and minimum during summer and monsoon respectively. It was understood from the study, ammonia could be acting as the limiting nutrient for phytoplankton growth, while the role of nitrate, nitrite, phosphate and silicate remained insignificant. At the time of diatom population proliferates there was a drop in the nutrient levels was observed during the study. The water current flowing from north to south during the northeast monsoon, nutrient rich fresh water discharged from Tamirabarani River influencing the nutrient dynamics in the fishing grounds that are ultimately increasing the nutrients concentration during northeast monsoon.

## Background

Phytoplankton diversity in the ocean may influence the functioning of marine ecosystems through overall productivity, nutrient cycling and carbon export (Goebel et al. 2013). The productivity of a specific water body depends on the amount of plankton present in the same water body (Guy 1992). The plankton growth and distribution depend on the carrying capacity of the environment, availability of the inorganic nutrients and the physico-chemical characteristics of the coastal waters. The nutrient contents in any coastal water determine its potential fertility (Harvey 1960), and the nutrient supply to phytoplankton subsequently enhances the species composition, population abundance, richness and rates of primary production (Hobday et al. 2006). The species composition and abundance of phytoplankton determine the zooplankton diversity and finally affects the fish production as indicated by Schroeder (1983). Variability in primary production may influence the fishery productivity and a strong link between phytoplankton and fisheries variability is proposed by Bainbridge and Mckay (1968) and Cushing (1975). All these factors in turn collectively support the fishery resources of coastal ecosystem. Any changes including depletion of nutrients and biological parameters would therefore affect the health of the coastal ecosystem and alternatively reduce the fish productivity. The knowledge of phytoplankton spatial variations of primary production, nutrient concentration and community structure is fundamental for the understanding of ecosystem dynamics (Bootsma and Hecky 1993). The health of coastal and marine ecosystems is depending upon the primary productivity and productivity potential of the coastal depends upon the primary producers. Although photosynthesis is a key component of the global carbon cycle, its spatial and temporal variability is poorly constrained observationally (Carr et al. 2006). Primary production has been performed by chlorophyll bearing plants ranging from the tiny phytoplankton to the giant kelps through the process of photosynthesis. Phytoplankton alone contributed to about 90.0 % of the total marine primary production (Satpathy et al. 2010). The physical process such as hydrodynamic conditions and current patterns are influencing the primary productivity and determining the phytoplankton’s distribution (Dickie and Trites 1983). Consequently, physical processes that can bring nutrients into the photic zone are of prime importance (Jayasiri and Priyadarshani 2007). *Chlorophyll ‘a’* (Chl-‘a’) is a unique parameter that influences the primary productivity of aquatic ecosystems and initiates the marine food chain. In marine ecosystem, Chl-a pigment is closely connected with photosynthesis and playing major role in fishery productivity in coastal and marine waters. Buttler and Tibbits (1972) reported that the Chl-‘a’ above 0.2 mg/l the presence of sufficient fish food to sustain a viable commercial fishery. The hydrographic conditions along the east coast of India undergo significant changes with seasons.

Nutrient concentrations in the coastal water column are the net result of removal processes and supply from rivers, municipal and industrial plant effluents, atmospheric deposition and sediment regenerations (Santschi 1995). Ions required for plant growth are known as nutrients and these are the fertilizers of the oceans (Duxbury and Duxbury 1999). Since the nutrients are life supporting factors of the marine ecosystems, inorganic substances nitrogenous nutrients (nitrate, nitrite, and ammonia) phosphorus and silicate are considered to be more important than others, as they are playing a key role in phytoplankton abundance, growth and metabolism (Raymont 1980; Grant and Gross 1996). The nutrient contents in any coastal water determine its potential fertility (Harvey 1960) and therefore investigations on nutrients distribution and behaviour in different coastal ecosystems are prerequisites for productivity evaluation. Considering these, the present study was conducted to understand the role of available inorganic nutrients in controlling the abundance and structure of phytoplankton populations in traditional fishing grounds of Tiruchendur coastal waters.

## Methods

### Description of the study area

**Fig. 1:**
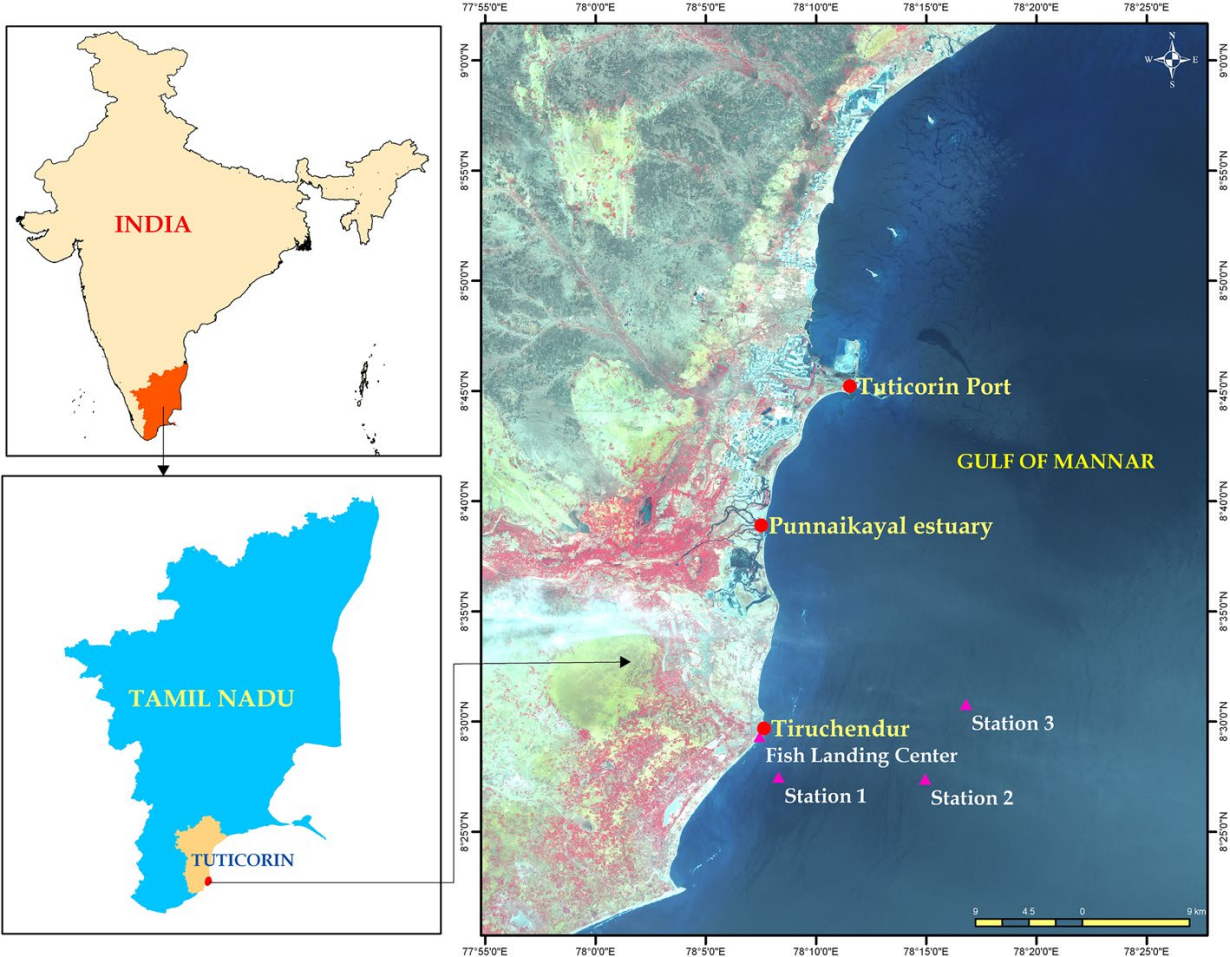
Map showing the study area

Tiruchendur is a coastal town (Lat: 8°.29′.19.1″N and Long: 78°.7′. 26.62″E) in the Thoothukudi District of Tamil Nadu. It is located between Thoothukudi and Kanyakumari and situated on the bank of Gulf of Mannar, Southeast Coast of India. Gulf of Mannar, located between the southeast coast of India and west coast of Sri Lanka is a unique marine environment, and rich in biodiversity. More than 3600 species of plants and animals inhabits Gulf of Mannar and is rightly referred as biologists’ paradise. Three traditional fishing grounds were chosen for investigation: Station 1 is located about 3.7 km from the shore at 10 m depth (Lat: 8°.27′.28.48″N Long: 78°.8′.18.48″E) (Fig. 1). This station is well known as a lobster and other crustaceans fishing ground with rocky bottom. Station 2 is located (Lat: 8°.27′.23.32″N and Long: 78°.14′.57.06″E) about 14.1 km from the shore at 30 m depth. The distance between Station 1 and 2 is about 10 km. Cuttlefish, pomfret, sardine fishes, Indian mackerel, seer fishes and other fishes are caught in this ground designated as Station 2 (Fig. 1). Station 3 is located (Lat: 8°.30′.46.2″N and Long: 78°.16′.48.15″E) about 17.3 km from the shore at 32 m depth and it is the important potential fishing ground for pelagic fishes such as sardine, anchovy, Indian mackerel, seer fishes and *Lates calcarifer* (Fig. 1).

### Data collection and methodology

#### Estimation of nutrients

To measure the distribution of inorganic nutrients of the fishing grounds (Stations 1–3) off Tiruchendur coastal waters, seawater samples were collected in 1 l pre cleaned polythene bottles at monthly intervals for 2 years. A fishing vessel made of Fibre Reinforced Plastic (FRP) was employed to collect the water samples throughout the study period. Samples were collected at early morning of the day between 6 a.m. and 9 a.m. Usually, sampling boat would start at 4 a.m.–5 a.m. from the shore and reach the fishing ground between 6.30 a.m. and 7.00 a.m. Niskin water sampler (1 l capacity) was used to collect the water sample and then transferred to the pre cleaned polythene bottles to estimate the nutrients. Collected samples were immediately kept in icebox and transported to the laboratory for the further analyses. The seawater samples were filtered using a Millipore filtering system through whatman membrane filter paper of 0.45 µ porosity. The quantity of the dissolved nutrients of ammonia-N, nitrite-N, nitrate-N, phosphate-P, silicate-Si present in the filtered water samples were determined, following the standard methods as described by Strickland and Parsons (1972).

### Estimation of Chl-a

The Chl-a concentration was calculated by adopting the following formula as described by Ramadhas and Santhanam (1996):

$${\text{Pigment}}\;\left( {{\text{mg/m}}^{3} } \right) = {\text{C}}/{\text{V}}$$

where V = volume of seawater filtered in 1 l. C = value obtained from the following equation:

$${\text{C}}\left( {{\text{Chl}} - {\text{a}}} \right) = 11.64\;{\text{E}}\;663 - 2.16\;{\text{E}}\;645 + 0.10\;{\text{E}}\;630$$

In order to eliminate the turbidity, the OD values of the acetone extracts (C value) was subtracted from absorbance at 750 nm.

### Estimation of primary productivity

The total primary productivity of the water column was estimated by the light and dark bottle method explained by Strickland and Parsons (1972). It was expressed in mg.C/m^2^/day and calculated by the following formula:

$${\text{Gross}}\;{\text{photosynthesis}}\;\left( {{\text{mgC}}/{\text{m}}^{3} /{\text{hr}}} \right) = \frac{{605 \times f\left[ { VLB - VDB } \right]}}{N \times PQ}$$

605 = The factor value used to convert oxygen value into carbon value. Where f = Dissolved oxygen (ml)/the quantity of sodium thiosulphate (ml) used in the titration. VLB = Volume of Light Bottle. VDB = Volume of Dark Bottle. N = incubation period in hours. PQ = photosynthetic Quotient = 1.25

### Enumeration of phytoplankton 

Phytoplankton samples were collected from the surface water column at monthly intervals by towing a phytoplankton net (0.35 m mouth diameter) made of bolting silk (No.30, mesh size 48 μm) attached with a calibrated digital flow meter (General Oceanics Inc, Florida). Thereafter phytoplankton samples were preserved in 4 % formalin in filtered seawater for the qualitative analyses and species level identification. For the quantitative analysis, the settling method as described by Sukhanova (1978) was followed. The cells counts and species were identified based on standard taxonomic keys according to Thomas (1977) and also as per the standard methods given in Desikachary et al. (1987), Anand et al. (1986). Before the microscopic analyses, samples were concentrated to 5 to10 ml by siphoning out the top layer with a tube covered with a 10 µm Nytex filter on one end. The required sample concentrates were transferred to a 1 ml capacity Sedgwick-Rafter counter and counted using a Nikon Binocular Dissection Microscope (Model: Nikon SMZ 1500) at 200× magnification. The total number of phytoplankton present in the collected sample was calculated by the following formula.

$${\text{N}}\; \frac{{ n \times {\text{v}}}}{V} \times 1000$$

where N is the total number of phytoplankton cells per litre of water filtered, n is an average number of phytoplankton in 1 ml of sample, v is the volume of phytoplankton concentrates, V is the volume of total water filtered. Species diversity index (Shannon and Weaver 1949), species richness (Gleason 1922) and evenness index (Pielou 1967) of phytoplankton were calculated by using the following respective formulae.

- ${\text{Shannon}}{\text{-}}{\text{Wiener}}\;{\text{diversity}}\; {\text{index}}\; \left( {{\rm H}^{\prime } } \right) = \mathop \sum \nolimits_{i = 1}^{s} Pi \log_{2} Pi$ where, S = total number of species, *Pi* = ni/N for the ith species, ni = number of individuals of a species in sample, N = total number of individuals of all species in sample. H′ = species diversity in bits of information per individual, where the value of H′ is dependent upon the number of species present, their relative proportions, sample size (N), and the logarithmic base. The choice of the base of logarithm is very important. In the present study, log_2_ has been used as per the practice in India.
- ${\text{Species}}\;{\text{richness}}\;\left( {\text{SR}} \right)\; = \;\left( {{\text{S}} - 1} \right)/{ \log }\;{\text{N}}$ where, S = number of species representing a particular sample, N = natural logarithm of the total number of individuals of all the species within the sample.
- ${\text{Species}}\;{\text{evenness}}\;{\text{or}}\;{\text{equality}}\;\left( {{\text{J}}^{\prime } } \right) = {\text{H}}^{\prime } /{ \log }_{2} {\text{S}}$ where, J′ = species evenness, H′ = species diversity in bits of information per individual, (observed species diversity). S = total number of species.

### Statistical analyses

To assess the relationship between phytoplankton population abundance and with various inorganic nutrients, Pearson’s correlation matrix was calculated by using statistical package SPSS (version 16.0). Two-way analysis of variance (ANOVA) for phytoplankton abundance for stations 1–3 was also calculated to understand the significance of differences of biodiversity indexes between temporal and spatial variations.

## Results

### Nutrients

**Fig. 2:**
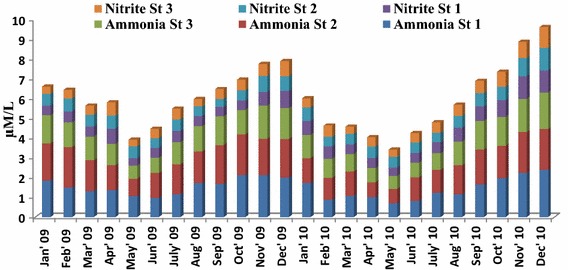
Monthly variation of ammonia and nitrite concentration in the fishing grounds

**Fig. 3:**
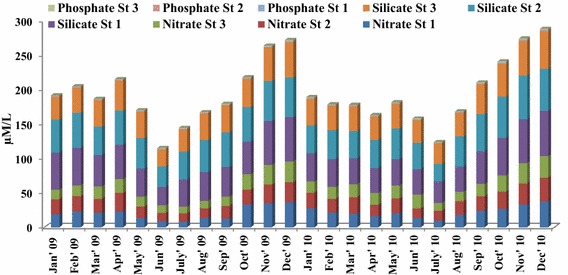
Monthly variations of phosphate, nitrate and silicate in the fishing grounds

**Fig. 4:**
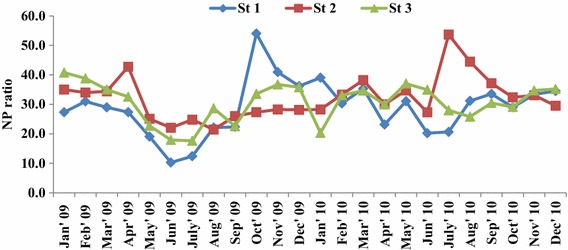
Monthly variations of NP ratio in the fishing grounds

Results of the inorganic nutrient distribution in the fishing grounds shows clear seasonal trend with maximum and minimum concentration observed during monsoon and summer season respectively. Two way ANOVA test revealed the significant temporal and spatial variation of nutrients in the fishing grounds (Tables 1, 2, 3, 4, 5). Ammonia species level significantly varied from 0.65 to 2.37 µM NH_4_^+^–N l^−1^ and minimum and maximum value were recorded in stations 3 and 1 respectively (Fig. 2). Nitrite concentration showed significant temporal and spatial variations ranged from 0.34 to 1.14 37 µM NO_2_–**N** l^−1^ and minimum and maximum value observed at station 1 (May, 2009) and station 2 (December, 2010) respectively (Fig. 2). Nitrate concentration temporally varied between 8.1 and 37.6 µM NO_3_–**N** l^−1^ during pre-monsoon and monsoon seasons respectively (Fig. 3). The inorganic phosphate concentration ranged from 0.3 to 1.29 µM PO_4_−P l^−1^ with peak value in monsoon season and low value in pre-monsoon recorded (Fig. 3). The highest silicate concentration (65.6 µM SiO_4_−Si l^−1^) was recorded at station 1 during December 2010 (Fig. 3) and minimum (24.53 µM SiO_4_−Si l^−1^) value was observed at fishing ground 3, during 2009 in the month of June. N/P ratio: The ratio of nitrogen-N to phosphorus-P was observed to range from 10.28 to 54 (Fig. 4) and showing seasonal similarity with inorganic nutrients.

**Table 1:** Two wav ANOVA test of ammonia-N

Source of variation	ss	df	MS	F	*P* value	F crit
Rows	1.360373	3	0.453458	57.41989	*P* < 0.05	4.757063
Columns	0.254617	2	0.127308	16.12065	*P* < 0.05	5.143253
Error	0.047383	6	0.007897			
Total	1.662373	11

**Table 2:** Two wav ANOVA test of nitrate–N

Source of variation	ss	df	MS	F	*P* value	F crit
Rows	0.170451	3	0.056817	45.72417	*P* < 0.05	4.757063
Columns	0.017693	2	0.008847	7.119483	*P* < 0.05	5.143253
Error	0.007456	6	0.001243			
Total	0.1956	11

**Table 3:** Two wav ANOVA test of nitrate–N

Source of variation		df	MS	F	*P* value	F crit
Rows	404.0372	3	134.6791	26.45367	*P* < 0.05	4.757063
Columns	21.18703	2	10.59351	2.080779	*P* < 0.05	5.143253
Error	30.54678	6	5.091129			
Total	455.771	11

**Table 4:** Two way ANOVA test of phosphate-N

Source of variation	ss	df	MS	F	*P* value	F crit
Rows	0.220512	3	0.073504	25.33896	*P* < 0.05	4.757063
Columns	0.042194	2	0.021097	7.272709	*P* < 0.05	5.143253
Error	0.017405	6	0.002901			
Total	0.280111	11

**Table 5:** Two way ANOVA test of silicate-Si

Source of variation	ss	df	MS	F	*P* value	*F*crit
Rows	572.175	3	190.725	47.71144	*P* < 0.05	4.757063
Columns	131.6594	2	65.82969	16.46784	*P* < 0.05	5.143253
Error	23.98482	6	3.997469			
Total	727.8192	11

### Chl-a and primary production

**Fig. 5:**
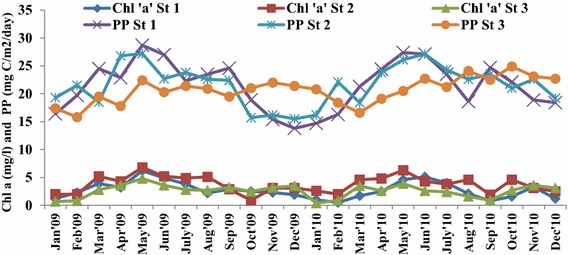
Monthly variation of Chlorophyll ‘a’ and Primary productivity in the fishing grounds

Chl-a concentrates revealed significant spatial and temporal variation (Table 6) from 0.4 to 6.8 mg/m^3^ with highest (Summer) at station 2 and lowest (post monsoon) at station 3 (Fig. 5). However, insignificant temporal and spatial variation of primary productivity was observed from 13.8 to 28.7 mg, C/m^2^/day (Fig. 5) with maximum and minimum during summer (May, 2009) and monsoon (December 2009) respectively in the fishing grounds (Table 7).

**Table 6:** Two way ANOVA test of Chloraphyll ‘a’

Source of Variation	55	df	MS	*F*	*P value*	*F *crit
Rows	8.52	3	2.84	10.97	*P* < 0.05	4.75
Columns	3.30	2	1.65	6.33	*P* < 0.05	5.14
Error	1.56	6	0.26			
Total	13.39	11

**Table 7:** Two way ANOVA test of primary productivity

Source of variation	SS	df	MS	F	*P* value	F crit
Rows	56.67213	3	18.89071	3.426085	NS	4.757063
Columns	2.116713	2	1.058356	0.191947	NS	5.143253
Error	33.08273	6	5.513789			
Total	91.87157	11				

*NS* not significant

### Phytoplankton abundance

**Fig. 6:**
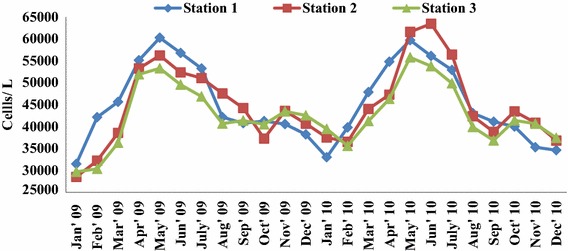
Monthly variation of phytoplankton density in the fishing grounds

A total of 73 species of phytoplankton were identified from the fishing grounds off Tiruchendur coast, revealed higher abundance in summer and low during Northeast monsoon in January months in all the sampling sites. Among the three stations, maximum phytoplankton abundance was recorded in station 2 followed by stations 1 and 3 (Fig. 6). Interestingly, phytoplankton is showing similar trend with zooplankton abundance in the study area. In station 1, the phytoplankton abundance ranged from 3.1 × 10^4^ to 6.02 × 10^4^ cells/l with minimum and maximum values in January and May 2009 respectively. The population abundance varied between 2.85 × 10^4^ (January 2009) and 6.34 × 10^4^ cells/l (June,2010) in station 2. At station 3, phytoplankton abundance from 2.9 × 10^4^ to 5.5 × 10^4^ cells/l with maximum and minimum values during May, 2010 and January, 2009 respectively. Two way ANOVA (Table 8) revealed significant temporal variation in phytoplankton abundance. Season based current pattern prevailing in Gulf of Mannar influencing the plankton productivity as reported by Selvin Pitchaikani and Lipton (2012) and Jagadeesan et al. (2013). The currents could be considered as playing the major role in the biological productivity of Gulf of Mannar.

**Table 8:** Two way ANOVA test of phytoplankton at Station 1, 2 and 3

Source of variation	ss	df	MS	F	*P* value	F crit
Rows	5.38E−08	3	1.79E−08	42.16409	*P* < 0.05	4.757063
Columns	14,420,662	2	7,210,331	1.694024	NS	5.143253
Error	25,537,995	6	4,256,332			
Total	5.78E−08	11				

*NS* not significant

#### Species composition 2009

**Fig. 7:**
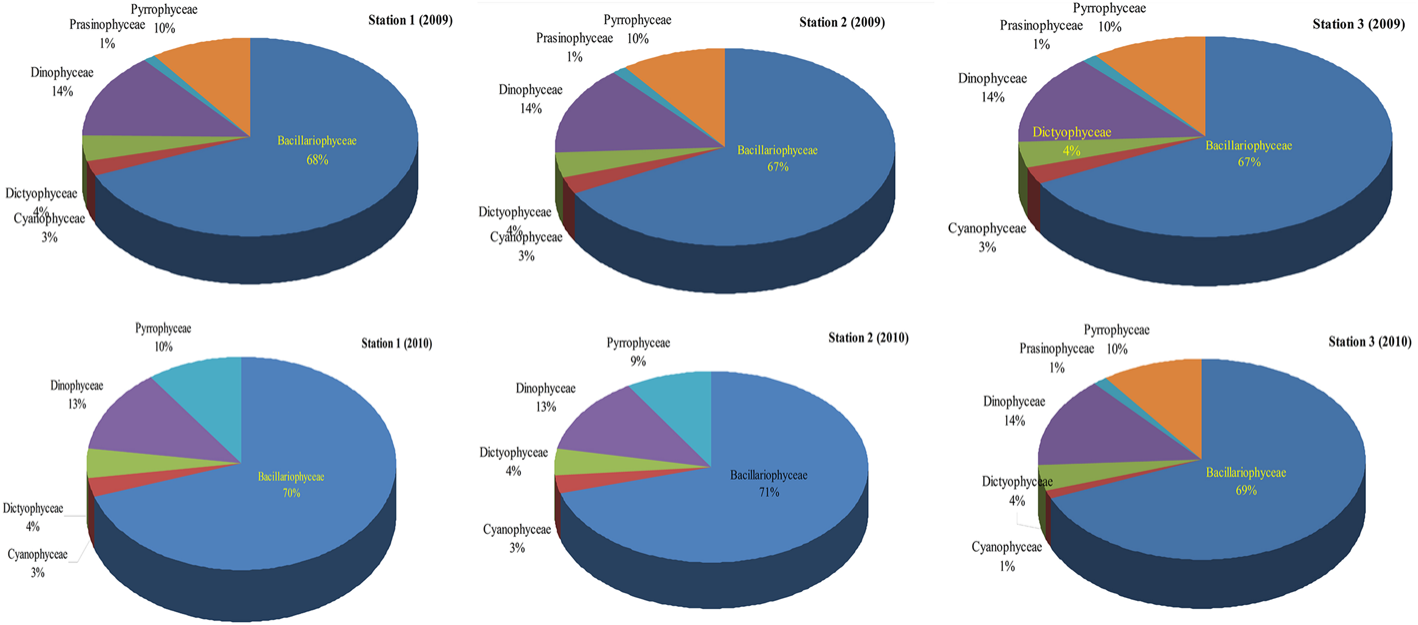
Composition of phytoplankton groups in the fishing grounds

Of the 73 species of phytoplankton recorded at station 1, 50 species were diatoms belonging to the family Bacillariophyceae with 68 %, 10 were dinoflagellates (14 %), 7 of them are Dictophyceae (4 %), 2 species of Cyanophyceae (3 %) and 1 species of Prasinophyceae (1 %) were observed (Fig. 7). At station 2, total number of 70 species, constituting of six classes were recorded during different seasons (Fig. 7). Among the six classes, diatoms were the dominant group with 47 species of Bacillariophyceae (67 %), 2 species of Cyanophyceae (3 %), 3 species of Dictophyceae (4 %), 10 species of Dinophyceae (14 %), one species of Prasinophyceae (1 %) and 7 species of Pyrrophceae (10 %) were recorded at station 2. Similar to station 1 and 2, in station 3, 50 species of Diatoms (67 %), 2 species of Cyanophyceae (3 %), 3 species of Dictophyceae (4 %), 9 species of Dinophyceae (14 %) 1 species of Prasinophyceae (2 %) and 7 species of pyrrophyceae (10 %) were observed (Fig. 7). *Cylindrotheca closterium, Ditylum sp, Skeletonema* sp. were dominant species among the *Diatom group. Dinophysis caudata, Dinophysis ovum* were dominant species among the Dinoflagellates.

#### Species composition 2010

The phytoplankton percentage composition of second year (during 2010) followed similar trend to that of the previous year (2009) (Fig. 7). In all the three stations, Diatoms formed the dominant group with percentage values of 70, 70.5 and 68.5 % were observed at stations 1–3 during 2010.

### Diversity indices

The species diversity indices Shannon–Wiener diversity (H′), species richness index (SR) and evenness index (J′) were considered as explanatory variables of dynamics of phytoplankton levels, which to some degree are interrelated and observed indices are given in Table 5.

### Shannon–Wiener diversity (H′)

At station 1, H′ varied between 3.678 and to 4.097, minimum and maximum were recorded in October (2009) and April (2009) respectively. The H′ values were found minimum (3.717) in January (2009) and maximum (4.084) in April (2009) for station 2. At station 3, diversity showing similar trend with station 2, and ranged between 3.788 and 4.142. The results of the calculated ecological Shannon diversity index of the fishing grounds reflected changes in the phytoplankton community structure because of seasonal impact (Table 9).

**Table 9:** Shannon diversity, species richness and species evenness at stations 1–3

	Species diversity			Species richness			Species evenness		
	St 1	St 2	St 3	St 1	St 2	St 3	St 1	St 2	St 3
Jan’09	3.741	3.717	3.788	0.9732	0.9713	0.866	0.7801	0.7622	0.975
Feb’09	3.788	3.802	3.792	0.9733	0.9725	0.8209	0.8029	0.7462	0.9739
Mar’09	3.827	3.969	3.962	0.9726	0.978	0.8089	0.7406	0.8147	0.9775
Apr’09	4.097	4.066	4.142	0.9807	0.9806	0.8863	0.8351	0.8578	0.9823
May’09	4.025	3.953	3.974	0.9778	0.9768	0.782	0.7775	0.7776	0.9766
Jun’09	3.898	3.73	3.821	0.976	0.9693	0.7365	0.7828	0.6947	0.9723
July’09	3.91	3.955	3.981	0.976	0.9787	0.8499	0.7924	0.856	0.9787
Aug’09	3.997	4.019	4.038	0.9793	0.9801	0.8859	0.8504	0.8829	0.9804
Sep’09	3.952	3.855	3.937	0.9764	0.9736	0.8008	0.7887	0.7155	0.9768
Oct’09	3.678	3.863	3.844	0.9642	0.9749	0.7785	0.6703	0.8069	0.9714
Nov’09	3.957	3.977	4.013	0.9782	0.9791	0.8646	0.8713	0.8894	0.9796
Dec’09	3.992	3.923	3.987	0.9798	0.9776	0.855	0.8882	0.8566	0.9795
Jan’10	3.936	3.845	4.023	0.9774	0.9747	0.8094	0.7648	0.7542	0.9787
Feb’10	3.847	3.904	3.969	0.9755	0.9764	0.8023	0.8077	0.7871	0.9777
Mar’10	3.99	4.009	4.046	0.9781	0.98	0.8529	0.7946	0.8889	0.98
Apr’10	4.044	3.982	4.016	0.9804	0.9788	0.8408	0.8644	0.8509	0.9794
May’10	3.964	3.986	4.043	0.9773	0.9794	0.8258	0.7979	0.868	0.9794
Jun’10	3.885	3.818	3.955	0.9763	0.9746	0.8418	0.7722	0.7715	0.9783
July’10	3.861	3.957	3.927	0.9756	0.979	0.8603	0.7787	0.8575	0.9784
Aug’10	3.927	3.979	3.981	0.9782	0.9794	0.9078	0.8188	0.8763	0.9796
Sep’10	3.934	3.879	3.959	0.9783	0.9752	0.8322	0.8662	0.7932	0.9773
Oct’10	3.842	3.856	3.901	0.9731	0.974	0.785	0.7397	0.7501	0.9744
Nov’10	3.897	3.936	4.041	0.9774	0.9787	0.8754	0.7821	0.8126	0.9803
Dec’10	3.912	3.949	4.046	0.9771	0.9784	0.8531	0.7812	0.837	0.9797

### Simpson species richness

At station 1, species richness ranged from 0.9642 (October, 2009) to 0.9807 (April, 2009). At station 2, species richness varied between 0.9693 and 0.9806 with minimum and maximum values recorded in June (2009) and April (2009) months respectively. At station 3, minimum species richness (0.7365) was recorded in June 2009 and maximum (0.9078) was recorded during August, 2010 (Table 9).

### Species evenness

At station 1, the species evenness varied from 0.6703 to 0.8882 with minimum and maximum values in October, 2009 and December, 2009 respectively. In station 2, evenness varied from 0.6947 to 0.8894 in which low value was recorded during June, 2009 and high value was recorded during November, 2009. A maximum evenness value of 0.9714 was observed in the month of October, 2009 at station 3 and the minimum (0.9823) was observed in April, 2009 (Table 9).

### Statistical analyses

In station 1, phytoplankton abundance exhibited positive correlation with Chl-a, (Table 10). The primary productivity was significantly correlated with phytoplankton abundance in station 1 and 2, however, it did not showed any significant correlation with phytoplankton at station 3. During the present study, biological factors (Chl-a, primary productivity and phytoplankton abundance) showed significant negative correlation with ammonia, nitrate, nitrite and silicate in station 1, but phosphate did not showed any correlation with biological factors (Table 10). Ammonia exhibited significant negative correlation with phytoplankton abundance in all the three stations at P < 0.001. At station 2, ammonia and nitrate showed negative correlation with biological parameters but nitrite showed insignificant correlation. At the same time, silicate did not show significant correlation with Chl-a and primary productivity but negatively correlated with phytoplankton in station 2 (Table 10). In station 3, ammonia showed significant negative correlation with phytoplankton abundance (Table 10).

**Table 10:** Inter correlation of biological parameters with nutrients at station 1, 2 and 3

	Ammonia	Nitrate	Nitrite	Phosphate	Silicate	Chl ‘a’	PP	Phytoplankton density
*Station 1*								
Chl ‘a’	−0.49*	−0.51**	−0.41*	−0.13 NS	−0.40*	1.00		
Primary productivity	−0.59**	−0.67***	−0.49*	−0.28 NS	−0.58**	0.73***	1.00	
Phytoplankton density	−0.78***	−0.62***	−0.54**	−0.30 NS	−0.64***	0.80***	0.80***	1.00
*Station 2*								
Chl ‘a’	−0.67***	−0.41*	−0.26 NS	−0.37 NS	−0.38 NS	1.00		
Primary productivity	−0.55**	−0.48*	−0.30 NS	−0.59**	−0.32 NS	0.54**	1.00	
Phytoplankton density	−0.66***	−0.49*	−0.35 NS	−0.43*	−0.46*	0.73***	0.70***	1.00
*Station 3*								
Chl ‘a’	−0.17 NS	0.27 NS	0.11 NS	0.31 NS	0.20 NS	1.00		
Primary productivity	0.26 NS	0.30 NS	0.47*	0.55**	0.37 NS	0.21 NS	1.00	
Phytoplankton density	−0.61**	−0.08 NS	−0.14 NS	0.07 NS	−0.22 NS	0.68***	0.27 NS	1.00

*NS* not significant

* *P* ≤ 0.05; ** *P* ≤ 0.01; *** *P* ≤ 0.001

## Discussion

The dynamics of phytoplankton are the net result of a complex interplay of physical, chemical and biological processes (Choudhury and Pal 2010). From the last few decades, there has been much interest to study different factors influencing the development of phytoplankton communities, primarily in relation to physico-chemical factors (Nielsen et al. 2002; Grenz et al. 2000; Elliott and Hemingway 2002). Overall, the succession pattern of phytoplankton communities in relation to nutrient variation will help to understand the ecosystem functioning as suggested by Magurran (1988) and Barnese and Schelske (1994). In the present investigation, a remarkable biodiversity changes occur at three fishing grounds with seasonal time scales. It was observed that, the dynamics of hydrological conditions combined with hydrographical and nutrient dynamics controlling the biological productivity of the fishing grounds. The grazing ability of the zooplankton also, determining the phytoplankton abundance of the coastal ecosystem. The zooplankton biomass also determined by the plankton feeding fishes. So, phytoplankton abundance is not only controlled by environmental conditions of the ecosystem but also by the predators of the food web.

The correlation analyses showed significant negative correlation of phytoplankton population with nutrient concentration. It is a common phenomenon that nutrient availability largely determines the diversity of phytoplankton. Fluctuations of primary productivity and nutrients are controlling the dynamics of phytoplankton. From the statistical perspective, it was understood that, ammonia could function as the limiting nutrient than nitrate in controlling the growth of the phytoplankton in the study area. Phosphate did not play any crucial role in the phytoplankton growth. Ammonia and silicate concentration are the essential for development and growth of the diatoms. Similarly, changes in physicochemical parameters of the water column due to various factors that significantly influence the phytoplankton population (Choudhury and Pal 2010). Whenever, diatom population flourished there, a drop in the nutrient levels also was observed in the surface waters. The fluctuation in nutrient concentration was mainly due to influx of fresh water from Thamirabarani river and monsoon rainfall.

About 14,709.2 Mcft (Million Cubic Feet) of fresh water discharged from Thamirabarani River at Punnaikayal estuary during northeast monsoon (Data provided by PWD, Government of Tamil Nadu, India, Tirunelveli). This water flux bringing nutrient rich water to the study area, further this nutrient rich water brought down to southern part of Gulf of Mannar due to northeast monsoon current. The southwest monsoon and northeast monsoon influenced current pattern also playing major role in determining the dynamics of the phytoplankton species diversity in the water column. During the northeast monsoon season, the East India Coastal Current (EICC) in the western Bay of Bengal flow equator ward and the main flow turn around Sri Lanka and transports low saline waters into the Arabian Sea. However, associated with the water current movement of East Indian Coastal Current, volume of water flow from Bay of Bengal enter into the Gulf of Mannar (Murty and Varma 1964; Rao et al. 2011; Jyothibabu et al. 2013), these phenomenon causes water exchange along with plankton biomass between Palk Bay and Gulf of Mannar were observed. Hence, the phytoplankton species recorded in the Palk Bay and Bay of Bengal has also been observed in Gulf of Mannar. Since water current flowing from north to south during the northeast monsoon, nutrient rich fresh water discharged from Thamirabarani River influencing the nutrient dynamics in the fishing grounds that are ultimately increasing the nutrients concentration during northeast monsoon. Comparatively, less rainfall and low discharge from Thamirabarani River of about 6702.63 Mcft was discharged into Gulf of Mannar during 2010. Consequently, less concentration nutrients were observed during 2010.

### N/P ratio

In aquatic systems, nitrogen or phosphorus is the most common limiting nutrient since other minerals required for growth may be present in abundance (Ryther and Dunstan 1971; Vince and Valiela 1973). Generally, nitrogen (N) limitation prevails in most of the marine ecosystems (Fisher et al. 1992; Howarth 1988). Changes in nutrient supply are often reflected in their ratios (Yin et al. 2001). Hence, the elemental ratios (nitrogen to phosphate) of coastal environment can be used as indicators of the status of nutrient loading or to predict productivity (De-Pauw and Naessens-Foucquaert 1991). The calculated N: P ratio could be used to predict the phytoplankton abundance and assemblages and to understand of the ecology of the phytoplankton (Jane 2007). Alternatively, phytoplankton productivity can be used to understand the fishery productivity and health of the coastal ecosystem. Generally, if the ratio of nitrogen and phosphorous exceeds 15:1, the available phosphorous is said to limit organic carbon production. If the N/P ratio value below 15:1, then nitrogen is the limiting nutrient (Redfield 1986). Based on the N/P ratio obtained from the present study, N/P ratio was remained above 15 in all the three stations except June, 2009 and July 2009 in station 1. According to the N/P ratio of the present study, phosphorous was the limiting nutrient in the fishing grounds. Generally, ammonia is known to suppress the uptake of nitrate and other nutrients by phytoplankton when its concentration exceeds 1 µg at N/l (McCarthy et al. 1982). Further, it was unique to observe that ammonia was remained above 1 µg at N/l in most of the time. Further, N/P ratio did not show any temporal and spatial variations during the course of the study. Even though, N/P ratio was above 15 level, based on the correlation study it was understood that, nitrogenous nutrients were the limiting factor for the phytoplankton productivity.

### Biological parameters

Low value of Chl-a observed during monsoon and post monsoon could be due to the dilution effect by the fresh water discharged from runoff and from riverine flow causing turbidity and less availability of light (Rajkumar et al. 2009; Thillai Rajasekar et al. 2010). In general, northeast monsoon become unfavourable for phytoplankton growth in coastal waters of Gulf of Mannar, due to precipitation and land drainage. The nutrients and light penetration in the water column are the important factors, influencing the productivity of marine ecosystem. The Chl-a bloom generally fully develops during June to July with high Chl-a contents in the Gulf of Mannar, Palk Bay and along the southern coast of Sri Lanka (Vinayachandran et al. 2004). Abdul Aziz et al. (2003), documented that high concentration of Chl-a pigment during summer season in Arabian Gulf waters. Therefore, it was understood that seasonal variation of Chl-a value in Bay of Bengal and Arabian Sea are showing the same trend.

The fluctuations of primary production coincided with Chl-a concentration of the euphotic zone, and influenced by the seasonal changes. As suggested by Sulochana and Muniyandi (2005), the reduced light intensity due to cumulus clouds formed during northeast monsoon exaggerated the primary productivity in the fishing grounds.

The high production of phytoplankton was observed in summer and pre-monsoon seasons, due to the stable and optimal conditions of salinity, sea surface temperature, light intensity (Sahu et al. 2012), euphotic depth of the water column, Chl-a production and optimum level of inorganic nutrients in the water column etc. prevailed during these periods.

It is a well-known phenomenon that temperature is a prime factor controlling the growth of the phytoplankton. Direct heating of the solar radiation causes high temperature and increasing the light penetration depth (i.e., euphotic zone). Collectively, all these parameters, supporting or inducing the phytoplankton growth during summer season (from April to June). Thereafter, productivity has been decreased gradually until monsoon. Low diversity and abundance were recorded during monsoon season due to the low salinity, low atmospheric temperature, sea surface temperature and minimum euphotic zone depth.

Since, the inorganic nutrients are the primary foodstuff of phytoplankton, high abundance of phytoplankton causes low nutrients level in the water column and vice versa. Similar values were reported from the Tranqubar-Nagapattinam coast by Sampathkumar (1992), Pondicherry coast (Ananthan 1995) and Vellar estuary (Perumal et al. 1999). Population abundance has been decreasing in the pre monsoon months through post monsoon months and after attaining low abundance at post monsoon in the month of January, again population abundance has been increasing gradually and attaining maximum abundance during summer months from April to June.

According to NEERI, (2004), diatoms and dinoflagellates contributed 70 and 30 % respectively in Palk Bay and Gulf of Mannar. However, the composition of dinoflagellates in the present study differed from that of the investigations conducted by NEERI during 2004. It is a common phenomenon that the diatoms are playing major role on contribution of primary productions in coastal waters and estuarine waters, which subsequently being transferred through copepods to fish (Madhu et al. 2007). *Biddulphia mobiliensis, Biddulphia rhombus, Chaetoceros densus, Coscinodiscus ecentricus, Skeletonema* sp. were the dominant species among the diatom group. Generally, diatom communities are influenced by environmental perturbations with monsoonal system that influence the niche opportunities of species (D’Costa and Anil 2010). Diatoms are the major phytoplankton group in coastal ecosystem, controlled by a complex suite of regulating factors.

The Shannon diversity index (H’) indicated the lowest phytoplankton composition stability during the monsoon season, whilst the highest heterogeneity and therefore stability of this structure was detected during summer, which corresponded to a high productive status of the Gulf of Mannar. The species richness index (SR) was highest during the summer, which correlated with a maximal species richness of the phytoplankton community (Pielou 1967). There is no clear trend of evenness was observed during the study period. During 2009, maximum species evenness was recorded during monsoon season in the month of June. However, during 2010, maximum evenness was recorded during post monsoon, summer and southwest monsoon season.

## Conclusion

In the present study, phytoplankton primary production and inorganic nutrients of the fishing grounds exhibited clear seasonal trend as influenced by prevailing monsoonal system in east coast of India. The diatoms are playing major role on contribution of primary productions in the fishing grounds off Tiruchendur coast, with more diatoms species were recorded at station 1 than station 2 & 3. The high production of phytoplankton was observed in summer and pre-monsoon seasons, which may be due to the stable and optimal conditions Chl-a production and inorganic nutrients in the water column prevailed during these periods. Phytoplankton abundance negatively correlated with nutrient concentration and nutrient availability chiefly determines the diversity of phytoplankton. Fluctuations of Chl-a, primary productivity and nutrients are controlling the dynamics of phytoplankton in the fishing grounds. It was understood that, ammonia could function as the limiting nutrient than nitrate in controlling the growth of the phytoplankton in the study area. Phosphate did not play any crucial role in the phytoplankton growth. At the time of diatom population flourished, there was a drop in the nutrient levels was observed during the study. The fluctuation in nutrient concentration was mainly due to influx of fresh water from Thamirabarani river and monsoon rainfall. The water current flowing from north to south during the northeast monsoon, nutrient rich fresh water discharged from Thamirabarani River influencing the nutrient dynamics in the fishing grounds that are ultimately increasing the nutrients concentration in the fishing grounds. Since the phytoplankton are the base of the marine food web, and transferring energy to secondary and tertiary levels, this study could be useful to understand the fishery potential of the environment for the sustainable utilization fishery resource and such information is critical for coastal resource management. Since this study formed the baseline data in the Tiruchendur coastal waters, it could be useful for the further research and for the sustainable ecosystem based fishery management.
